# Laparoscopic Repair Modality of Perforated Peptic Ulcer: Less Is More?

**DOI:** 10.7759/cureus.30926

**Published:** 2022-10-31

**Authors:** Lubomír Tulinský, Demet Sengul, Ilker Sengul, Ján Hrubovčák, Lubomír Martínek, Markéta Kepičová, Anton Pelikán, Peter Ihnát

**Affiliations:** 1 General Surgery, Faculty of Medicine, University of Ostrava, Ostrava, CZE; 2 General Surgery, University Hospital Ostrava, Ostrava, CZE; 3 Pathology, Giresun University Faculty of Medicine, Giresun, TUR; 4 Endocrine Surgery, Giresun University Faculty of Medicine, Giresun, TUR; 5 General Surgery, Giresun University Faculty of Medicine, Giresun, TUR; 6 General Surgery, Univerzita Tomáše Bati ve Zlíně, Zlín, CZE

**Keywords:** pathology, surgical pathology, histopathology, laparoscopy, emergency, acute abdomen, suture dehiscence, laparoscopic repair, perforated peptic ulcer

## Abstract

Perforation, per se, presents the most serious complication of peptic ulcer disease with a mortality rate that cannot be underestimated. Surgery is the only treatment option, which can be performed laparoscopically or via conventional laparotomy. The present study aimed to compare the short-term outcomes of laparoscopy and laparotomy techniques in the surgical treatment of peptic ulcer perforation. A retrospective study design was structured to compare the perioperative and short-term postoperative outcomes of 102 patients who had undergone laparoscopic and conventional repair of the perforated peptic ulcer over a six-year interval (January 1, 2016, to December 31, 2021). Of these, 44 (43.1%) had undergone laparoscopic repair while 58 (56.9%) had surgical repair via conventional laparotomy. The operative time and length of hospital stay were comparable in both subgroups (p=0.984 and p =0.585). Nevertheless, 30-day postoperative morbidity was significantly higher in the open surgery subgroup (75.9% vs. 59.1%, p= 0.032). The risk of relaparotomy was similar in both study subgroups; however, suture dehiscence as a reason for surgical revision was significantly more frequent in the laparoscopic subgroup (13.6% vs 3.4%). Of note, the mortality rate in the laparoscopic group of patients was 13.6%, and in the laparotomy group 41.4%. The laparoscopic approach to peptic ulcer perforation is the procedure of choice for low-risk patients. Conventional surgery seems to be associated with a significantly higher incidence of severe postoperative complications and mortality. However, the higher mortality in these patients is probably related to their worse initial clinical condition.

## Introduction

Peptic ulcer (PU) disease is one of the most common diseases of the stomach and duodenum. The worldwide incidence of uncomplicated PU is approximately 90 cases per 100,000 inhabitants per year. The basic etiological factors are the presence of *Helicobacter pylori,* non-steroidal anti-inflammatory drugs (NSAIDs), smoking, and stress [1]. Bleeding occurs in about 50% of PU cases and perforation in 10%. The worldwide average incidence of acute PU perforation is approximately nine cases per 100,000 inhabitants [2]. The main risk factors for acute ulcer perforation are women over 60 years of age, smoking, alcohol consumption, and the use of NSAIDs.

Mortality of PU perforation is generally around 10-40% [3,4], but varies significantly, depending on the patient's age and condition before admission to the hospital. In addition, patient risk can be classified according to well-known general scoring systems (eg, the American Society of Anaesthesiologists (ASA) score, the Acute Physiology and Chronic Health Evaluation (APACHE) II score). For the patient with PU perforation, specific classification systems such as the Boey score, the Hacettepe score, the Jabalpur score, and the PU perforation score can be used [5]. Currently, the Boey score is considered the most accurate and simplest system to determine the risk of mortality in patients with acute PU perforation [6,7].

The only treatment option for PU perforation is a surgical suture of perforation, which can be performed via conventional laparotomy or using minimally invasive surgery techniques. The choice of surgical approach (laparoscopy versus laparotomy) usually depends on the surgeon's preferences and the patient's clinical condition before surgery. In recent decades, there has been a growing tendency to use a minimally invasive approach in this acute indication. As such, some recent studies have revealed that laparoscopic perforation repair (LR), compared to surgical repair via conventional repair (CR) by laparotomy, may be associated with a lower number of postoperative complications and accelerated patient recovery [8,9]. In contrast, a recent meta-analysis found no differences in postoperative morbidity and mortality between the mentioned surgical approaches [10].

From a technical point of view, the suture of the perforation presents the main critical point of the procedure. The laparoscopic suture cannot always be tightened as thoroughly as an open surgical approach would allow, which can increase the risk of suture dehiscence in the postoperative period. It is reasonable to believe that the higher risk of suture dehiscence will be associated with higher postoperative morbidity/mortality.

The main aim of the present study was to compare the short-term outcomes of laparoscopic and conventional surgery techniques in the surgical treatment of PU perforation. We postulate that the perceived higher risk of suture dehiscence in patients undergoing laparoscopic repair of the PU perforation could be associated with significantly higher postoperative morbidity and mortality.

## Materials and methods

This retrospective cohort study was conducted in the Department of Surgery, University Hospital Ostrava, Ostrava, Czech Republic. All cases that had undergone an LR or CR of the PU perforation over six years (January 1, 2016, to December 31, 2021) were assessed for study eligibility. The surgical approach depended on the age and clinical condition of the patient. The elderly and patients with comorbid diseases were indicated for laparotomy. Last but not least, cases with incomplete data in the hospital information system were excluded from the present study design.

Before surgeries, all cases were administered a double dose of broad-spectrum antibiotics, and after surgical procedures, antibiotic therapy was continued therapeutically. Regardless of the surgical approach, the PU perforation was always sutured with several individual absorbable stitches and an omentoplasty was performed subsequent to the surgical suture of the perforation. Afterwards, a thorough abdominal lavage was performed with a diluted iodine solution, followed by drainage (one to two silicone drains placed around the suturated locations and in the pelvis) and a nasogastric tube was inserted into all the patients in order to collect gastric contents. LR was performed using the three trocars (in the supraumbilical, left mesogastric, and right mesogastric areas), and open repair procedures were performed using upper middle laparotomies.

Demographic and clinical data from all study patients (age, sex, body mass index (BMI), ASA classification, location of the ulcer), type of surgery revision surgery, and 30-day postoperative morbidity were obtained from medical records data. Their preoperative clinical conditions were assessed according to the Boey score (Table 1) and the duration of perforation before admission was defined as the period between the onset of acute symptoms or the acute exacerbation of chronic pain and admission to the hospital. Systolic blood pressure <100 mmHg was considered a preoperative shock and systemic heart, lung, liver, or kidney disease, cancer, and diabetes were considered concomitant severe medical illnesses [7]. Postoperative complications were assessed according to the Clavien-Dindo classification [11].

**Table 1:** The Boey score

Boey score	
1 point	Duration of perforation >24 h
1 point	Preoperative shock
1 point	Concomitant severe medical illness

The obtained data were analyzed using descriptive statistics. The mean, standard deviation, and t-test for quantitative values were used for the statistical description of the set. The Chi-square test was used for categorical values and a level of significance of α=0.05 and p-values <0.05 were considered statistically significant.

## Results

The study incorporated a total of 102 patients after surgical repair of the PU perforation. There were 44 (43.1%) patients after LR and 58 (56.9%) patients after PU perforation repair with upper middle laparotomy. The basic demographic and clinical characteristics of all studied patients are presented in Table 2. There were 44 (43.1%) females and 58 (56.9%) males with a mean age of 65.8 ± 14.8 years (ranging from 32 to 93 years, p = 0.041) and a BMI of 24.2 ± 4.8 kg/m2. In total, 20 (19.6%) cases were preoperatively classified as ASA I-II, while 46 (45.1%) were classified as ASA III and 36 (35.3%) as ASA IV-V. Gastric ulcer perforation was detected in 62 (60.8%) cases, while duodenal perforation was detected in 34 (33.3%) patients and gastroduodenal location for perforation in six (5.9%) patients. After admission to the hospital, 27.5% of our study patients had Boey score of 0, 29.4% of the patients had 1, 33.3% of patients had 2, and 11.8% of our patients had 3.

**Table 2:** Demographics and clinical data of study patients

	Laparoscopy, n=44	Laparotomy, n=58	p value	Total, n=102
Age, years, mean±SD	62.3 ± 16.5	68.5 ± 13.0	0.041	65.8 ± 14.8
Gender, n (%)			0.309	
Female	22 (50.0%)	22 (37.9%)		44 (43.1%)
Male	22 (50.0%)	36 (62.1%)		58 (56.9%)
BMI, kg/m^2^, mean±SD	23.8 ± 3.2	24.5 ± 5.7	0.410	24.2 ± 4.8
ASA, n (%)			0.020	
I-II	10 (22.7%)	10 (17.2%)		20 (19.6%)
III	22 (50.0%)	24 (41.4%)		46 (45.1%)
IV-V	12 (27.3%)	24 (41.4%)		36 (35.3%)
Ulcer location, n (%)			0.006	
Gastric	20 (45.5%)	42 (72.4%)		62 (60.8%)
Duodenal	22 (50.0%)	12 (20.7%)		34 (33.3%)
Gastroduodenal	2 (4.5%)	4 (6.9%)		6 (5.9%)
Boey score, n (%)			0.044	
0	18 (40.9%)	10 (17.2%)		28 (27.5%)
1	12 (27.3%)	18 (31.0%)		30 (29.4%)
2	12 (27.3%)	22 (37.9%)		34 (33.3%)
3	2 (4.5%)	8 (13.8)		12 (11.8%)

Regarding the Boey score and the ASA classification, patients undergoing LR of PU perforation had a better general health status after admission to the hospital. As such, 40.9 % of the study patients who underwent LR had a Boey score of 0 while only 4.5% had a Boey score of 3. In contrast, 13.8% of the cases that underwent open repair had a Boey score of 3 while 17.2% had a Boey score of 0. Herewith, the differences in the Boey score between the study subgroups were statistically significant (p=0.044). In addition, short-term postoperative outcomes are presented in Table 3 with an average surgery time of 58.9±23.3 min without a significant difference between subgroups (p=0.984). The mean postoperative hospital stay was 12.9±7.8 days (range, 3-32 days, LR 12.5±7.5 days, OR 13.3±8.2 days) without a significant difference between the subgroups (p=0.585). The 30-day postoperative morbidity rate was 68.6%. Postoperative complications were experienced by 59.1% who underwent LR and 75.9% who underwent CR, with statistical significance (p=0.032).

**Table 3:** Intraoperative and postoperative outcomes of study patients

	Laparoscopy, n=44	Laparotomy, n=58	p value	Total, n=102
Surgery time, min, mean±SD	59.0 ± 20.5	58.9 ± 25.5	0.984	58.9 ± 23.3
Hospital stay, days, mean±SD	12.5 ± 7.5	13.3 ± 8.2	0.585	12.9 ± 7.8
30-day postoperative morbidity, n (%)	26 (59.1%)	44 (75.9%)	0.032	70 (68.6%)
Revision surgery, n (%)	6 (13.6%)	10 (17.2%)	0.767	16 (15.7%)
Suture dehiscence, n (%)	6 (13.6%)	2 (3.4%)	0.007	8 (7.8%)
Clavien-Dindo classification, n (%)				
Grade 0	18 (40.9%)	14 (24.1%)	<0.001	32 (31.4%)
Grade I-II	12 (27.3%)	12 (20.7%)		24 (23.5%)
Grade III-IV	8 (18.2%)	8 (13.8%)		16 (15.7%)
Grade V (postoperative mortality)	6 (13.6%)	24 (41.4%)	0.001	30 (29.4%)

According to the Clavien-Dindo classification, mild complications (grade I-II) were noted in 24 (23.5%) patients, and serious complications (grade III-V) in 46 (45.1%). Patients after laparoscopic surgery showed a higher incidence of mild complications compared to patients after conventional surgery (27.3% vs. 20.7%), but also a higher percentage of an uncomplicated postoperative course (40.9% vs. 24.1%). On the other hand, patients, after open surgery, had a significantly higher incidence of complications classified as grade V. Differences between study subgroups regarding the severity of postoperative complications were statistically significant (p<0.001). 30-day postoperative mortality was 29.4% in our study group. Mortality was significantly higher in the conventional subgroup of patients compared to the laparoscopic one (41.4% vs 13.6%, p=0.001). In total, 16 (15.7%) study cases had to undergo redo surgery, including six (13.6%) from the laparoscopic subgroup and 10 (17.2%) from the conventional without statistical significance (p=0.767).

All redo surgery in the laparoscopic subgroup of cases was caused by suture dehiscence with associated localized/generalized peritonitis. Herein, the perforation was re-sutured by utilizing the laparotomy in two cases and laparoscopically in four. In the laparotomy subgroup of 10 redo surgery, only two were performed due to suture dehiscence: one for bleeding, the other for intraabdominal infectious complication (the collection of infected fluids). The difference in the rate of suture dehiscence as a reason for redo surgery was statistically significant (p=0.007).

## Discussion

The basic procedure for the treatment of PU perforation is a suture of the relevant anatomic location. In the past, this procedure was performed predominantly using laparotomy. Some of these concerns no longer seem like optimistic management for the mentioned condition. LR for perforation was first described in 1990 [12] and since then we have seen a significant onset of laparoscopy even in severe acute indications. Wright et al. reported an LR rate of just 2.6% in the population of 5361 cases during the study period 2007-2010 [13]. In a study of 4210 patients, Davenport et al. reported an increase in laparoscopy rates from 4.5% in 2010 to 11.4% in 2016 [14]. Recent studies report an LR of PU perforation of 10-50% [15-17]. The sudden increase in minimal access surgery in the acute abdomen cannot be underestimated [18-20]. Of note, the present study has a laparoscopy ratio of 43%, which is consistent with the aforementioned findings.

The laparoscopic approach in the therapeutic management of PU perforation has been assessed in several studies [16,21]. LR has shown similar or better short-term results than open repair in terms of postoperative morbidity, mortality, operative time, and hospital stay; however, leakage can be significantly higher [22]. Our preliminary results have demonstrated that the suture dehiscence rate after LR is three times higher than the dehiscence rate after CR. In contrast, we did not confirm higher postoperative morbidity and mortality in the laparoscopic subgroup of our patients. The postoperative mortality in open PU perforation repair was even three times higher than that in LR. This finding is probably the consequence of the input characteristics of our study subgroups, id est, better general health status after hospital admission in the laparoscopic subgroup of cases. The Boey score was used to assess the initial clinical condition of all cases of the present study [7]. Many authors consider the Boey score to be the most accurate and simplest scoring system to determine the mortality risk in patients with acute PU perforation [5,6,23,24]. Our cohort revealed that surgeons selected patients with lower Boey scores (Boey 0-1) for laparoscopic PU perforation repair. Patients with a higher score (Boey 2-3) had undergone predominantly CR. Therefore, the cases selected for LR were less at risk than patients undergoing laparotomy. This results in a significant difference in mortality between our study subgroups (postoperative mortality of CR was three times higher than that of LR).

Hereinabove, in terms of postoperative morbidity, LR can be considered equivalent to CR. However, some authors suggest that the perforation cannot be sutured sufficiently during laparoscopy, leading to a higher risk of revision surgery for suture dehiscence [25]. This assumption was already refuted by Zhou et al. [26] in their study in 2009 and later by Wilhelmsen et al. [27] in 2015 without significant differences in the revision rates after LR and CR. However, in LR, the reason for the reoperation was suture dehiscence at 100% (compared to 20% in CR, p=0.007). According to our outcomes, peptic ulcer suture dehiscence seems to be significantly more common in patients undergoing LR of PU perforation.

An LR can be performed with or without an omental patch. In our study, we did not address the impact of this approach. Recent studies have shown that in terms of leakage rate and surgical outcome, the maneuver to cover an omental patch in LR did not show additional advantages compared to simple closure alone [28]. In our cohort, an omentoplasty has been performed in all cases, so we cannot comment on its effect on leakage. Differences in patient age, ASA classification, and Boey scores between study subgroups were statistically significant, which is related to the nonrandomized design of the study. Surgeons have chosen the surgical approach (laparoscopy vs. laparotomy) according to the patient's condition. Elderly patients and patients with higher ASA and Boey scores are at increased risk, so in our study, surgeons have opted for a safer procedure, LR. Another point to consider is that in this era, delayed diagnosis in emergency conditions leads to more-inflammatory alterations and irreversible cyto- and histopathologic changes in the relevant tissues and organs [29]. To this end, emergency surgery remains significant in the aforementioned era, worldwide [30].

Limitation

The main limitation of our study is the retrospective and nonrandomized study design. However, the study has a sufficient number of patients, and it was carried out in a single institution by experienced surgeons. Standard surgical and postoperative procedures were maintained for all patients, guaranteeing a reduction in the risk of skewed results.

## Conclusions

The laparoscopic approach to PU perforation is the procedure of choice for low-risk cases (Boey score 0-1). Preoperative determination of mortality risk using the Boey score is accurate and advantageous to the choice of surgical approach. There were statistically significant differences in age, ASA and Boey score between the study groups, suggesting that lower-risk patients are generally indicated for LS. According to our data, the risk of suture dehiscence is significantly higher in patients undergoing LR. However, CR seems to be associated with a significantly higher incidence of severe postoperative complications and mortality. The higher mortality of these cases is probably related to their worse initial clinical condition. *Fide sed cui vide* (trust but be careful of whom you trust). This issue merits further investigation.
